# Perspectives of people who use drugs on implementing overdose response technologies in acute care settings: a qualitative study

**DOI:** 10.1186/s13722-025-00636-0

**Published:** 2025-12-13

**Authors:** Avnit Dhanoa, Dylan Viste, William Rioux, Boogyung Seo, Maria Vasquez, Stephanie Vandenberg, Chris Anhorn, S. Monty Ghosh

**Affiliations:** 1 Department of Medicine, Faculty of Medicine & Dentistry, University of Alberta, 2J2.00 Walter C Mackenzie Health Sciences Centre, 8440 112 St NW, Edmonton, AB T6G 2R7 Canada; 2 Department of Medicine, Cumming School of Medicine, University of Calgary, Calgary, AB Canada; 3 Department of Internal Medicine, Faculty of Medicine & Dentistry, University of Alberta, Edmonton, AB Canada

**Keywords:** Overdose response technologies, People who use drugs, Acute care, Harm reduction

## Abstract

**Background:**

People who use drugs (PWUD) face many barriers in healthcare settings. Illicit substance use during hospital stays, including solitary use in bathrooms, is prevalent, leading to a higher risk of overdoses. In this study, we examine the views of PWUD on implementing novel strategies such as overdose response technologies (ORTs) into acute care and explore the perceived acceptability, impacts, and barriers of these interventions.

**Methods:**

We used convenience sampling to recruit 10 participants from hospitals and addiction medicine clinics, and semi-structured interviews were conducted. The interviews included an explanation of the five main types of ORTs relevant to acute care settings (hotlines, applications, overdose buttons, reverse motion detectors, and wearables), followed by questions where the participant had to critically evaluate whether each ORTs would be effective for each scenario. Open-ended coding and thematic analysis were used, and themes were derived from the data as it was reviewed.

**Results:**

Participants acknowledged the advantages and potential risks of integrating ORTs into acute care. It was recognized that ORTs could help improve the relationships between PWUD and healthcare providers, reduce mortality rates in hospital bathrooms, and provide peer support during hospital stays. PWUD highlighted privacy concerns, logistical barriers, and stated that ORTs can also negatively impact their relationships with healthcare providers due to stigma.

**Conclusion:**

Although many participants felt that incorporating ORTs would be an advantage to their care within hospitals, our study also highlighted implementation barriers and broader policy changes that need to be addressed. Working towards addressing such barriers and changes can allow ORTs to be the next tool to help mitigate barriers faced by PWUD.

**Supplementary Information:**

The online version contains supplementary material available at 10.1186/s13722-025-00636-0.

## Introduction

People who use drugs (PWUD) are hospitalized at a much higher rate than the general population, with an average of 15 hospitalizations each day in Canada being attributed to opioid-related poisonings [1, 2]. Illicit substance use in clinical settings is an ongoing concern and many factors can drive substance use in acute care, such as the cravings being overwhelming during their stay, self-reported pain not being validated or managed by healthcare providers, severe unmanaged withdrawal symptoms, and an attempt to avoid negative experiences like loneliness while hospitalized [3].

Research has found that implementation of hospital-based safe consumption sites could address the gaps within patient care for this demographic [4]. Safe consumption sites allowing patients to use illicit substances have reduced the number of fatal overdoses within hospitals and reduced risk of blood-borne infections [4, 5]. However, there are a limited number of in-hospital safe consumption sites in North America, and individuals reported fearing criminalization and stigma if they accessed this service, including from hospital staff [4, 5]. Additionally, a study in an urban medical centre in the United States reported 357 overdoses in the bathroom from 2016 to 2018 and a study in England reported 236 overdose-related deaths during hospital admission across a decade [6, 7]. As such, Overdose Response Technologies (ORTs) have been proposed to address the ongoing lack of harm reduction services within hospital settings and the significant number of fatal overdoses within these environments [6, 8].

ORTs are defined as technology that reduces the risk of a drug poisoning event, particularly among those who use substances alone. These technologies take on a variety of forms, including hotlines, smartphone applications, reverse motion detectors, overdose response buttons, and wearable technologies [8]. Overdose response hotlines, such as the National Overdose Response Service (NORS), connect PWUD with individuals with lived experience to monitor the call. Callers create an emergency response plan with operators, and if the individual becomes unresponsive, they will dispatch an appropriate responder to administer naloxone [9]. Application based services, such as the Digital Overdose Response System (DORS), involve an emergency alarm that has to be disabled at regular time intervals, and failure to do so will dispatch emergency medical services to their geographic coordinates [9]. Other technologies include wearable technology and fixed devices, such as buttons and reverse motion detectors. Overdose buttons can be described as wireless buttons that can contact a designated response team when pressed, and reverse motion detectors monitor an individual’s movement and timing once the system is activated in locations such as bathrooms [10, 11].

Previous literature has explored healthcare providers’ perspectives on implementing such ORTs in acute care settings [12]. While it was found that these technologies could be beneficial to reduce stigma, improve rapport building, and increase the safety of PWUD, they need to be implemented as a part of broader harm reduction interventions with an emphasis on holistic care [12]. Despite these findings, to our knowledge, there is no published research on incorporating ORTs into hospitals from the perspectives of PWUD. In this study, we examine their perspectives on implementing ORTs into acute care (inpatient and emergency settings).

## Methods

The reporting of this study was guided by the Consolidated Criteria For Reporting Qualitative (COREQ) Research framework [13].

### Design

As an initial exploration into the potential uses of ORTs in acute care settings, we conducted qualitative interviews with PWUD who had experienced a hospital stay in the past year. Semi-structured interviews were chosen so that both relevant or unexpected topics could be explored in proper depth. An interview guide was created by W.R. guided by Proctors framework, which focuses on developing and applying strategies to improve evidence-based interventions [14].

### Recruitment

10 participants were recruited from May 2024 to November 2024. The inclusion criteria were as follows (1) be a resident of Canada, (2) over 18 years of age, (3) be able to verbally consent and communicate in English, (4) have access to a phone or video chat, (5) have a history of illicit substance use, and (6) have accessed hospital services as a patient within the past year. Participants’ substance of choice ranged from methamphetamine, fentanyl, heroin, cocaine, etc. and demographics are provided in Table 1. Recruitment was conducted through convenience sampling via research posters in hospitals and two addiction medicine clinics in Alberta. Clinicians and health care staff at the addiction clinics also verbally shared the study with patients but emphasized that participation was optional. Individuals received a $30 honorarium after participating.

### Interviews

Interested participants contacted the research contractor group, ThreeHive Consulting, who arranged virtual 1-hour interviews by video chat or phone with two female researchers, both with master’s degrees and experience in qualitative interviewing. The researchers followed a standard process: they explained the five main types of ORTs relevant to acute care settings (hotlines, apps, buttons, reverse motion detectors, and wearables), then asked participants to critically evaluate whether each ORT would be effective for specific scenarios (e.g., for people who prefer to smoke or people in a hospital bed). Participant’s comparisons between each ORT is provided in Table 2. The interviewers had no prior relationships with participants. They continued conducting interviews until there were no more emerging themes, thus determining thematic saturation and the sample size. They did not conduct repeat interviews and did not return transcripts to participants for comments or corrections. An interview guide can be found in the supplementary material.

### Qualitative analysis

The interviewers transcribed and analyzed the original interviews using Dedoose software. They applied open-ended coding and thematic analysis to explore novel phenomena, deriving themes as they reviewed the data. Whenever possible, ThreeHive Consulting staff linked each code to the specific ORTs in question (e.g., “Hotlines – unable to speak in a shared room”). After completing the initial coding, one researcher from the main team (AD) reviewed the codes and sorted them in Microsoft Excel into thematically similar categories (e.g., “Privacy is essential for ORTs”). Once arranged thematically, the other authors (DV, MG, BS) reviewed these categories and provided final suggestions. The team also conducted triangulation and shared results with key stakeholders, including clinicians and other PWUD, to ensure the themes aligned with their experiences.

## Results

**Table 1:** Participant demographics

Demographics	Total
**Total**	10
**Age (years)**	
Mean Age $\:\pm\:$ SD (Minimum Age – Maximum Age)	38 $\:\pm\:$ 7.64 (26–52)
**Gender**	
Male	4 (40%)
Female	4 (40%)
Gender fluid, Transgender Female	1 (10%)
Two-Spirit	1 (10%)
**Province**	
Alberta	8 (80%)
Ontario	2 (20%)
**Urban/Rural**	
Urban	9 (90%)
Rural	1 (10%)
**ORT preference when asked to pick one service they would like to see most**	
NORS	3 (30%)
DORS	3 (30%)
Wearables	2 (20%)
Reverse Motion Detectors	1 (10%)
Buttons	1 (10%)

**Table 2:** Comparisons of overdose response technologies

Overdose Response Technologies	PROS	CONS
Hotline based Overdose Response e.g. National Overdose Response System	- Provides connection and peer support by connecting to operators with lived experiences. - Provides timely care.	- Requires speaking out loud, compromising privacy in shared rooms. - Dependent on phone access.
Smartphone Application based Overdose Response Systems. Eg. Digital Overdose Response System / Lifeguard	- More discrete and private monitoring. - Provides timely care.	- Technology access and literature barriers. - Less personal connection without peer support. - Alarm sound will take away from discreteness.
Reverse Motion Detectors	- Increases safety where solitary substance use occurs. - Reduces overdose mortalities. - Potential to reduce stigma if it can help anyone experiencing a medical emergency in a bathroom (e.g. heart attack), not just PWUD.	- False alarms could occur - Potential for stigma as privacy is compromised. - Fear of being monitored.
Wearables	- Discrete and attracts minimal attention from others in shared rooms.	- It could make PWUD easily identifiable if the design is well known. - and labels as “overdose related”
Overdose Buttons	- Familiar technology, similar to hospital buttons - If placed in every room, could help with fatalities while protecting from stigma.	- Slow response times from healthcare staff - May increase stigma if it’s labeled visibly for overdose response - Requires the individual who presses the button to be awake and alert to press the button which may not be possible before an overdose (although they can press the button to alert staff to check on them later on if they’re using substances).

### Section 1: Relationships between PWUD and healthcare teams

Most participants believed implementing ORTS would strengthen their relationship with healthcare providers by being better understood and enhancing their sense of safety. Some believed that implementing ORTs could help shift providers’ perspectives on substance use, leading to more compassionate care.

*I think it would help broaden their own understanding of addiction*,* and maybe open their minds a little bit*,* and maybe it would bring patients and physicians or health care workers closer together*,* when dealing with the addiction. - P6*,* female*

*It would make me feel hopeful*,* just knowing the provider’s telling me about these interventions that could happen*,* right? Having the*,* yes*,* having the support of them in any way would be helpful*,* hopeful. - P11*,* male*

Additionally, participants shared that if healthcare providers openly discussed available resources, they would feel supported rather than judged for their substance use. Several participants felt that simply having healthcare providers initiate a conversation about overdose technologies and taking the time to discuss safety measures is a step towards feeling valued within hospitals.

*I would feel relieved. A little bit of relief that someone is taking the time to talk to me… they want people to have access to it*,* to these resources*,* to these tools*,* and to safety in this toxic drug crisis. - P10*,* non-binary*.

*But I definitely would feel*,* really*,* a lot safer and a lot*,* like*,* more at ease knowing that*,* like*,* OK*,* they care about me*,* they genuinely do care about me - P7*,* female*

Although most participants believed that ORTs could have a positive impact on their relationship with healthcare providers, many also expressed concerns that their implementation might elicit feelings of fear and stigma from healthcare providers. For many individuals, this concern was rooted in previous negative experiences within hospital settings, where they felt judged, dismissed, or treated unfairly due to their substance use history.

*The judgement and the lack of compassion sometimes when it comes from health practitioners. Because they look at it and say*,* you shouldn’t be doing this. Don’t do this. I can’t help you do this. It’s your own fault for doing this. -P6*,* male*

Some participants stated that they wouldn’t feel comfortable utilizing ORTs if healthcare providers were unaware of their history of substance use, as they wanted to avoid the stigma often associated with their struggles. They feared that if providers knew about their substance use, they might receive lower-quality care or be treated with less compassion.

*I mean it would depend on the reason why I’m at the hospital. If I was in the hospital setting for something completely different and they had no idea about my addiction*,* then no. I don’t think I would feel comfortable doing it*,* again*,* because of the stigma around labels and all that. - P5*,* female*

*Some nurses who may or may not have problems with junkies like me just might say something*,* whether even meaning to under their breath or not… but if it’s just going to bring more judgment*,* more discrimination*,* more whatever else*,* even if it’s not even intentional. - P10*,* non-binary*

However, while it was still recognized that bias in healthcare exists, not every practitioner discriminates against PWUD and it depends on the person.

*That’s a deep question*,* because at the end of the day it comes down to that individual person and how much training has gone into these people in regards to harm reduction and how they view it and what their biases are. - P8*,* female*

### Section 2: Concerns about privacy

Several participants mentioned that shared rooms would deter them away from certain overdose technologies, due to minimal privacy. Individuals emphasized that having to speak out loud would discourage them from NORS due to fears of being overheard and judged for their substance use. Shared hospital rooms also led participants to prefer wearables and reverse motion detectors, where you are not overheard.

*As I said before*,* if I’m not able to speak freely*,* I would feel more comfortable having something*,* having a way that I could respond to a situation like this*,* by being monitored without – I’d like to still be able to respond*,* but if I can’t talk because of the current situation*,* then I like the option. - P10*,* non-binary*

*Depending on how it’s labelled*,* the wearables*,* the motion detectors*,* absolutely. If NORS was the default option and well you have to be talking well*,* you are immediately*,* you are immediately disclosing to everybody else*,* and that may not make you feel safe anymore. - P10*,* non-binary*

Participants also expressed that services such as DORS allows for such privacy and discreteness, which influences their desire to use such applications. Some participants explained how if they shared a room with an individual who did not use substances, they may feel embarrassed accessing ORTS, thus making overdose buttons and wearables a better option.

*Depends. If they’re going through the same thing as me*,* yes. If not*,* then I probably would not*,* I’d be too shy. - P7*,* female*

*Like it’s more – like an alarm going off like that could just be my phone ringing or somebody. Nobody around me would know what was going on*,* and like – but that comes from shame and guilt*,* and I guess a lot of addicts do feel that. - P8*,* female*

Additionally, several participants stated that they would not want to put someone else in an uncomfortable situation, especially in a shared room. It was expressed that if buttons or wearable devices were visibly associated with substance use or overdose in the hospital room, they would no longer be discrete and could lead to feelings of stigma and judgment. To encourage use, individuals preferred these resources to be available in all rooms without drawing attention to their purpose, ensuring privacy and reducing the risk of being labeled.

*Because I think that something that monitors your heart rate and blood system*,* to the point of*,* like*,* dropping*,* so you’re overdosing*,* or just they need help in general is a good thing. And you wouldn’t have to be surrounded by drug use or stigma around it. - P4*,* female*

*The only reason why it would make me so hesitant to press it*,* is if there was like a big sign around it that said*,* like*,* Overdose Button. - P8*,* female*

### Section 3: Increase safety in bathrooms

Participants emphasized that because hospital bathrooms allow for privacy, they are more inclined to use substances alone in stalls. Thus, participants also highlighted that hospital bathrooms are where overdoses are more likely to occur due to solitary substance use. As a result, having reverse motion detectors could reduce overdose-related fatalities and ultimately save lives.

*I think that would be really helpful in a hospital setting*,* because if I was in a hospital room*,* and if I was going to use it*,* I would use it in the bathroom. So that would be the place where I am more likely to overdose*,* 110%. - P8*,* female*

*Because of judgment I think*,* judgment of normal*,* of people who don’t understand substance use. I think that’s my thing that I jump to*,* and then I get all caught up in that if there’s people who are there just to treat their common cold or whatever*,* and I’m sitting there shooting up*,* I just*,* I don’t know if I could do that. But in the bathroom*,* I could do that. - P11*,* male*

Individuals expressed how reverse motion detectors can expand beyond PWUD and help anyone who may experience a medical emergency in a bathroom setting, such as individuals who have a heart attack in the bathroom. Several participants noted that reverse motion detectors could assist individuals with various medical emergencies, not just overdoses. Because of this broader application, these devices are less likely to single out individuals experiencing an overdose, potentially reducing stigma. In contrast, technologies like overdose buttons are designed specifically for overdose situations.

*Actually*,* I think that would be a better way to go about it instead of the button*,* because then you may not be labelled*,* as you said*,* you could be … Whereas these sensors*,* it doesn’t matter if you use the button or not*,* either way they’re going to go off*,* and then that’s also helpful for other people with other medical conditions. Right? So maybe a person might not be using*,* but a person might*,* you know*,* drop a heart attack or who knows. So that would be helpful for other people as well. - P5*,* female*

### Section 4: Access to peer support

When asking participants about which ORTs they would be more comfortable using, peer support was a common theme. Many indicated they would access NORS to receive virtual peer support during their hospital stay. They saw these technologies as a safety net, fostering meaningful connections with individuals who had lived experience with substance use, helping create a non-judgmental space.

*There’s a real lack of that. And somebody who’s been through it is going to have that compassion*,* is going to have that empathy*,* is going to actually look after that person because they care about that person knowing that they need to be loved and so they can learn how to love themselves. -P5*,* male*

*It gives a level of comfort. And they know what you’re talking about*,* you don’t have to explain the details of how much they understand already. I think it’s very*,* very important that they have that peer value. -P9*,* female*

Participants described peer support as a crucial element in both harm reduction and recovery, helping them feel understood and less isolated. The ability to connect with someone who had navigated similar experiences created a sense of trust and encouragement, which they felt was often missing in traditional healthcare settings.

*The opposite of addiction is connection. Having a connection with somebody is going to have a much better chance of keeping me*,* - one*,* safe*,* and two*,* - maybe helping me to propel myself out of addiction and into recovery… The biggest one that I can suggest is having peer support available It’s just like there isn’t – it’s so much harder to make a connection with people that don’t understand what you’re going through. And when accessing resources*,* when I have the opportunity*,* I have always leaned on peer-lead - P5*,* male*

### Section 5: Logistical barriers

Some participants expressed concerns that the long response times already present in hospitals would be a barrier to implementing ORTs such as buttons. Many shared that this concern stemmed from their personal experiences of waiting extended periods to receive care within the healthcare system, such as when a patient presses a call button.

*Knowing what I know about how long it takes the nurse to get here*,* no. - P2*,* two-spirited*

*Because I mean*,* I’ve hit that nurse button before*,* and I haven’t seen a nurse for*,* like*,* half an hour*,* an hour*,* which is*,* you know. So I would assume that this would be a newly implemented thing where it would be crucial that they come and check on that patient in five minutes*,* right? -P5*,* female*

However, participants also indicated that they would be more likely to use NORS and DORS, as these options provide timely support.

*The nurse could be busy and something could go wrong*,* so having that app and that app in the shared room is definitely beneficial I believe. - P9*,* female*

## Discussion

This study aimed to examine the perspectives of PWUD on implementing virtual overdose technologies in acute care settings. The findings suggest that PWUD view ORTs to be a valuable tool within hospitals, as they can help reduce mortality, have the potential to improve relationships with healthcare providers, and provide virtual peer support. However, PWUD also revealed that barriers such as lack of privacy and stigma within the healthcare system would limit them from utilizing such resources. These are elements to improve upon when considering overdose technologies for acute care. These implications are valuable when assessing the barriers and potential impacts to integrating ORTs into hospital settings to improve care for PWUD.

Our findings highlight the lack of privacy as a barrier to implementing ORTs in acute care. This is valuable information with regards to expanding current harm reduction strategies to prioritize discreteness (such as texting options) and for tools like buttons and wearables, as it is crucial to design them with privacy in mind. It is imperative to note that while these services may be more discrete, there may be challenges in locating clients at risk for overdose, as these services leverage geolocation software, which may pose a challenge in large hospital settings. Furthermore, these services are traditionally directly connected to emergency medical services, which may not have jurisdiction to provide interventions within healthcare centers. One possible solution to this challenge is having the NORS operator contact the hospital unit when there is an overdose, thus initiating an emergency response. Additionally, some hotline services such as NORS have recently begun offering a texting service to address potential privacy concerns [15]. Therefore, increasing education and awareness about the option to text a peer operator with NORS could improve accessibility and utilization while ensuring the privacy of PWUD who may want to use this resource during their hospital stays.

Solitary substance use in public bathrooms has been long documented in literature and participants in the study highlighted the widespread use of substances in hospital bathrooms, as they emphasized that these spaces offer a sense of privacy and protection [16]. Many participants suggested that installing reverse motion detectors could help reduce the risk of fatal overdoses. Aligning with previous research, when hospital staff were asked about anti-motion alarm systems, they have also expressed that anti-motion alarm systems could be a tool to prevent overdoses, though high costs and technological challenges such as false alarms may be barriers [16]. Despite this, they acknowledged that a false alarm is an acceptable alternative to an overdose [16]. To reduce false alarms and optimize battery life, research has shown that detectors can use a low-power presence detection mode to trigger respiration monitoring only when a person enters the stall, and will re-check for human presence before sending an overdose alert [6]. When integrating ORTs such as reverse motion detectors in acute care settings, it is crucial to consider the technological design to ensure that it is minimizing the number of false alarms to reduce healthcare providers burnout and ensure privacy and confidentiality of the individual.

A common theme that arose in our results was how the integration of ORTs, such as NORS, would allow for more access to peer support. Connecting individuals to others with lived experiences has been seen as a method to help overcome system barriers within hospitals faced by PWUD, such as stigma and system rigidity [17]. Integrating ORTs within acute care settings can connect PWUD with others with lived experiences and provide a sense of community and security, especially if there is a lack of peer support workers at the hospital. Previous research on ORTs has demonstrated that these technologies provide mental health support during periods of isolation, and having ORTs as a tool in acute care can allow for connection to help mitigate such feelings [18].

Research indicates that healthcare providers often hold more negative attitudes toward PWUD compared to patients with other chronic illnesses [19]. Consistent with our findings, participants described experiencing stigma from their healthcare providers during their hospital stay and how this may instill fear when using ORTs. Addressing this issue is crucial for improving the implementation of ORTs while ensuring PWUD feel safe and respected in acute care. These findings demonstrate the need to enhance trauma-informed addiction training for healthcare providers particularly in academic and professional education, as research demonstrates the positive impact of educational interventions of patient care for substance use and stigma reduction [19]. Furthermore, emerging literature advocates for expanding hospital-based care for PWUD to include a broader range of evidence-based interventions, such as opioid agonist therapy, sterile supply programs, naloxone kit provision, and access to social and peer support services [20, 21]. Such interventions have been associated with improved engagement in addiction care, reduced overdose risk following discharge, and further promote a holistic, equitable approach to treatment that addresses both clinical and social needs [20, 21]. Collectively, these strategies highlight the importance of integrating harm reduction within acute care systems to enhance care for PWUD. In addition, reflecting on the implementation of hospital-based safe consumption sites may provide valuable lessons for integrating ORTs in acute care settings. Research on hospital safe consumption sites highlights the critical role of staff education, engagement, and policy support in fostering acceptance of harm reduction initiatives and ensuring effective practice within clinical environments [22]. Similar approaches, such as staff training and the inclusion of harm reduction principles in institutional policy, could facilitate the successful adoption of ORTs in hospitals, helping to reduce stigma and promote patient-centered care [10, 22].

Although the recruitment for this study was conducted across Canada, the majority of participants resided in Alberta. This geographic concentration may have influenced the findings, as healthcare experiences can vary between provinces. As a result, the experiences captured in this study may not fully represent the perspectives of individuals from other regions with different healthcare structures, limiting the studies generalizability. Future research could study a diverse demographic across Canada to assess the role ORTs can play in acute care. Furthermore, even though we had a sufficient number of participants for a qualitative study, the sample size could also be a limitation [23]. Additionally, further research can focus on assessing the logistical and systemic challenges associated with its implementation in acute care settings, including for hospitals in rural areas.

## Conclusion

This study aimed to understand the perspectives of PWUD towards implementing ORTs in hospitals. Although many participants felt that incorporating ORTs would be an advantage to their care within hospitals, our study also highlighted implementation barriers and broader policy changes that need to be addressed. Working towards addressing such barriers and changes can allow ORTs to be the next tool to help mitigate barriers faced by PWUD and allow for safer care.

## Supplementary Information

Below is the link to the electronic supplementary material.

Supplementary Material 1

## Data Availability

No datasets were generated or analysed during the current study.
