# Supplementary material for: Perspectives of people who use drugs on implementing overdose response technologies in acute care settings: a qualitative study
Source: Addict Sci Clin Pract. 2025 Dec 13;21:6. doi: 10.1186/s13722-025-00636-0 (PMC12805751; doi:10.1186/s13722-025-00636-0)
Supplement: Supplementary file 1 — Supplementary Material 1 [file 13722_2025_636_MOESM1_ESM.docx]

**NOTES FOR THE QUALITATIVE TEAM**

Thank you for your great work thus far, the following interview guide looks to gauge people's opinions on using a variety of virtual overdose monitoring services in a variety of settings.

The inclusion criterion are as follows:

Participants must be greater than 18 years of age

Participants must be able to verbally consent and speak in English

Participants must have a history of using substances

Participants need to be in one of the following categories:

- Has accessed hospital services (emergency department or in-patient services within the previous year)

We are aiming to have 15 participants total and aim to have significant overlap across categories while still maintaining a diversity of population views. Please only ask the questions that pertain to each individual's settings (ie: if they have only accessed acute care and in-patient services in the last year only ask that section from the following questions, if multiple categories ask multiple sections).

START INTERVIEW HERE:

Thank you for agreeing to talk with me today. We would like to talk to you about something called virtual supervised consumption services. These services can help keep people safe when they use drugs and can be especially helpful for people who use alone. As you know, when individuals use some drugs, especially opioids, there can be a risk of fatal overdose. Virtual supervised consumption services can be a telephone service or a smartphone app overdose response button and reverse motion detector. Each of these methods monitors people who are using substances and activates emergency services or an emergency response plan if the person using becomes unresponsive. We will go into a bit more detail on each of these later. Today we want to learn about what you think about these services specifically within acute care settings like hospitals. Evidence has shown that some people will continue to use drugs in hospitals resulting in a risk of overdose deaths.

We understand that some of the questions we need to ask may touch on sensitive topics. Your comfort and well-being are important to us. Before we proceed with the interview, we'd like to ask a few eligibility questions. Please feel free to answer with a simple "yes" or "no" to the following:

[Note for interviewer: Participants MUST say yes to all questions to take part in an interview]

|  | Yes | No |
| --- | --- | --- |
| Are you over the age of 18? |  |  |
| Can you communicate fluently in English? |  |  |
| Do you have a history of using illicit substances within the past year? |  |  |
| [For the questions below, participants just need to answer “yes” to one of these locations] |  |  |
| Have you accessed hospital services within the previous year as a patient? |  |  |

There are no right or wrong answers to my questions. We are hoping the conversation won’t take more than 60 minutes. Please know that I do not have a personal interest in virtual supervised consumption services in general, so please feel free to speak openly and honestly.

Due to the large number of questions we are aiming to get through today, I may cap our question response time to a couple of minutes. Once we’ve passed the time cap, I will politely ask if we can wrap up your response and move on to the next question, if that’s okay with you?

To ensure I capture all the valuable insights shared today, I would like to record our discussion with your permission. Direct quotes may be used from the recordings for research purposes, however, any identifying information will be removed and you will be anonymized. It will not be possible to tell who said what. Is this okay with you?

[If no, we cannot continue with the interview]

Your participation in this interview is completely voluntary and you have the right to stop from the discussion at any time. All identifying information will be removed from the transcript and any notes I take. You have the right to not answer questions, so please let me know if you would like to skip through any. Participating in no way affects any services you currently receive through Virtual Overdose Monitoring Services (VOMS) or may want to access in the future. Please feel free to speak openly and honestly, there are no right or wrong answers.

Do you have any questions? Is it okay to proceed?

Ok great just a few screening questions:

What is your age?

Have you heard of overdose detection technologies or virtual overdose monitoring services before?

Have you ever used VOMS before, if so, which one?

1. If you have ever used any of these services, would you say that they have changed your behavior regarding substance use? If yes, how have these services impacted your life?

# SPECIFICS

## NORS/BRAVE:

Let us start by diving into some of the tools more specifically and then I will ask you some more broad questions. To start with, let's talk about NORS. NORS or the National Overdose Response Service is a direct line to someone with lived or living experience of substance use who will virtual observe you you while you use. In the event of an emergency and if the person was unresponsive, the peer operator would call someone to alert them of a suspected overdose. In a hospital this could be a nurse or unit clerk, at a harm reduction housing facility, it could be a front desk worker or peer worker, or at a camp it could be a designated responder.

***Acute care***

1. Would you feel comfortable accessing this type of service in a hospital environment?
   1. Why or why not?
2. Would you use this service in a hospital environment?
   1. Why or why not?
   2. how might these services benefit you?
   3. Can you think of reasons you might be hesitant to use these services?
3. Do you think that the peer support /connection to someone with lived experience of substance aspect would be valuable in the healthcare setting?
   1. Why or why not?
4. Would you still consider using this in a shared room?

## DORS/Lifeguard

The DORS/Lifeguard apps are phone applications with a countdown timer, failure to refresh this timer triggers an alarm that gets louder and louder. If you don't respond to the alarm, emergency medical services (paramedics from outside the hospital) would be notified to respond.

***Acute Care***

1. Would you feel comfortable accessing this type of service in a hospital environment?
   1. Why or why not?
2. Would you use this service in a hospital environment?
   1. Why or why not?
3. Within a hospital setting, how might these services benefit you? Can you think of reasons you might be hesitant to use these services?
4. Would you still consider using this in a shared room?

## BUTTONS

Overdose response buttons in hospital settings would be small devices anchored to walls. If using in a hospital, clicking the button would alert a healthcare worker to check on you in 5 minutes.

***Acute Care***

1. Would you feel comfortable using a button response type of service in a hospital environment?
   1. Why or why not?
2. Within a hospital setting, how might these services benefit you? Can you think of reasons you might be hesitant to use these services?
3. Would you use this service in a hospital environment?

## REVERSE MOTION DETECTORS

These are sensors in bathrooms and other small spaces that are always active or activate as soon as you enter, and that would trigger an emergency response if someone does not move for more than 1 minute.

1. If you had a choice of using drugs in a bathroom with a reverse motion detector and one without which would you choose and why?

**Acute Care**

1. Within a hospital setting, how might these services benefit you? Can you think of reasons you might be hesitant to use these services?

***WEARABLE DEVICES***

*These are devices that people can wear like smart watches or finger probes which monitors a person’s heart rate and oxygen levels. If they have a reduction in heart rate or oxygen levels, such as during an overdose, it would activate an alert and emergency response.*

**Acute Care**

1. If you planned on using opioids in a hospital, would you feel comfortable asking for a wearable device like this?
2. Within a hospital setting, how might these services benefit you? Can you think of reasons you might be hesitant to use these services?

#

# GENERAL ACUTE

**The following questions are of a sensitive in nature. Feel free to not answer them. If you’re feeling triggered, please do let us know and we can provide you with resources and supports to contact. We have someone on standby should you need to support you.**

1. Can you tell us more about the circumstances in which you use substances? Have you been hospitalized before and wanted to use in hospital? How have harm reduction services, or the lack of harm reduction services potentially impacted your hospital stay?
2. Would using these services in hospitals make you feel safer while using substances?
3. Would you be worried about getting kicked out of hospitals if you used these services?
4. Between these technology-based services and a physical supervised consumption site at a hospital, what would you choose and why?
5. Do you think people who use drugs in hospitals will find these services stigmatizing?

- Why or why not?

1. Do you think these services would help to improve the relationship between people who use drugs and the healthcare system especially when it comes to stigma?

- Why or why not

1. Do you think this would help to build relationships between people who use drugs and healthcare providers like nurses and doctors?
   1. Why or why not?
2. Do you think these services would change how healthcare workers interact with PWUD? If so how?
3. If a healthcare worker were to discuss these options with you how would that make you feel?
4. How concerned are you about privacy around your substance use in hospital, and how do you think these services could promote or worsen your privacy?
5. What if any recommendations would you make regarding implementing these services in hospitals?

# FOR ALL RESPONDENTS

1. If you had to pick one service you would like to see most out of the ones I had just mentioned, which one would it be and why?
   1. Hotline based overdose services like NORS
   2. Timer based apps like DORS and Lifeguard
   3. Reverse motion detectors
   4. Wearable devices
2. How might these services improve to better support your health and safety?
   1. How do you think using these services has impacted the risk of using substances alone
3. Some of these services require a phone or a smartphone to be able to access, do you think that people would find it inequitable/ unequal to offer these services in hospitals if some people can’t access them?
4. Have you experienced any issues with wait times when using some of these virtual services especially hotline-based services? If yes, how did the situation affect you? Were you discouraged to use these services in the future?
5. Are there any additional services you think are missing from some of the services I mentioned previously?
6. Is there anything else I haven’t asked about that you would like to share?

# DEMOGRAPHICS:

Only a few more quick questions. The following questions will be used to help us understand if virtual supervised consumption services affect different people and groups in different ways. Please note no information that identifies you, including the following information, will be reported with any of the other information you provided in the report. Again, you do not have to answer any of the questions if you do not want to.

1. **What is your current gender identity? (Select one)**

**2. How old are you? _____________(years)**

**3. In what province do you currently live?**

- **Do you live in a rural or urban area?**

**4. What types of drugs do you use/ have you used?**

**5. What is your preferred method of using drugs? (Select one)**

Smoking/inhalation

Snorting

Injecting

Swallowing

Drinking

**6. Have you ever used drugs in a hospital environment?**

Thank you for doing this. You are eligible for $40 via e-transfer. Would you be able to share your email with us so we can arrange for a money transfer? It will not be associated with the answers you have given today. Thank you so much again for your help.

Appendix A: Support Services

Should the participant feel discomfort or triggered by the content in the conversation, please provide the following resources for them reach out to:

- For URGENT medical concerns, including overdose, please dial 911.
- To receive URGENT crisis support in Calgary, please call the Calgary Distress Centre at 403-266-HELP (4357).
- Outside of Calgary, please call 211 for assistance or Crisis Services Canada at 1-833-456-4566 (1-866-277-3553 in Quebec).
- To be connected with addictions services in Alberta, please call Health Link Alberta at 811.

Appendix B: If the participant uses substances during the interview

- Tell the participant that our interview protocol states we must take their address and will call 9-1-1 in the case of an overdose
- If the participant **agrees to sharing their address and calling 9-1-1**, take their address, and continue the interview. Monitor the participant for signs of overdose (not responding, slurred speech, not making sense, slow/erratic breathing, choking sounds, [video call only] blue tint to skin (for lighter skinned people) or grey/ashen tint (for darker skinned people)
  - Check-in with the participant, ask them if they are good to continue at multiple points throughout the interview. If the above symptoms arise and the participant does not respond to calling their name, call 9-1-1 and let the operator know the participant’s address and that they are unresponsive (do not use term ‘overdose’ for stigmatizing reasons)
  - If the participant **does not agree to sharing their address OR you are not comfortable in the situation,** tell the participant that you must end the interview. Give the participant the phone number for NORS (888-668-6677). Let the participant know that they are welcome to reschedule the interview at another time
